# A Threshold Switching Selector Based on Highly Ordered Ag Nanodots for X‐Point Memory Applications

**DOI:** 10.1002/advs.201900024

**Published:** 2019-04-02

**Authors:** Qilin Hua, Huaqiang Wu, Bin Gao, Meiran Zhao, Yujia Li, Xinyi Li, Xiang Hou, Meng‐Fan (Marvin) Chang, Peng Zhou, He Qian

**Affiliations:** ^1^ Institute of Microelectronics Tsinghua University Beijing 100084 China; ^2^ State Key Laboratory of ASIC and System School of Microelectronics Fudan University Shanghai 200433 China; ^3^ Department of Electrical Engineering National Tsing Hua University Hsinchu 30013 Taiwan

**Keywords:** Ag nanodots, cross‐point, one‐selector‐one‐resistor (1S1R), selectors, threshold switch

## Abstract

Leakage interference between memory cells is the primary obstacle for enlarging X‐point memory arrays. Metal‐filament threshold switches, possessing excellent selectivity and low leakage current, are developed in series with memory cells to reduce sneak path current and lower power consumption. However, these selectors typically have limited on‐state currents (≤10 µA), which are insufficient for memory RESET operations. Here, a strategy is proposed to achieve sufficiently large RESET current (≈2.3 mA) by introducing highly ordered Ag nanodots to the threshold switch. Compared to the Ag thin film case, Ag nanodots as active electrode could avoid excessive Ag atoms migration into solid electrolyte during operations, which causes stable conductive filament growth. Furthermore, Ag nanodots with rapid thermal processing contribute to forming multiple weak Ag filaments at a lower voltage and then spontaneous rupture as the applied voltage reduced, according to quantized conductance and simulation analysis. Impressively, the Ag nanodots based threshold switch, which is bidirectional and truly electroforming‐free, demonstrates extremely high selectivity >10^9^, ultralow leakage current <1 pA, very steep slope of 0.65 mV dec^−1^, and good thermal stability up to 200 °C, and further represents significant suppression of leakage currents and excellent performances for SET/RESET operations in the one‐selector‐one‐resistor configuration.

## 1 Introduction

Cross‐point (X‐point) memory technology is a promising building block for the next‐generation nonvolatile memory and neuromorphic computing applications.^1^, ^2^, ^3^ Resistive memory is considered to be capable of extremely high densities and ultralow power consumption due to its simple two‐terminal structure and reducing device size as small as 4*F* ^2^ (*F* is the minimal feature size). Indeed, a large, densely packed X‐point memory array will be employed to meet the requirements for these emerging applications. However, X‐point array based on resistive memory devices is typically suffering from sneak path currents from neighboring cells during write or read operations,^4^, ^5^, ^6^ which would seriously hinder the device operations and large‐scale integration. Many types of selector devices with integration of a resistive memory at each X‐point cell have been studied to deal with the sneak path problem. Currently, transistors are typically used as selector devices in one‐transistor‐one‐resistor (1T1R) configuration,^7^, ^8^ but these three‐terminal structure devices inevitably compromise the scaling advantages of X‐point architecture due to large feature size of transistor.^6^ Instead, two‐terminal selector devices are more prone to X‐point integration, which is considered as one‐selector‐one‐resistor (1S1R) configuration.^4^, ^5^, ^6^ Additionally, complementary resistive switches^9^, ^10^ and self‐rectifying devices^11^, ^12^ are also proposed to deal with the sneak path issue.

Previous work has reported a number of selectors mainly based on nonlinear threshold switching materials or tunneling barrier structures, including ovonic threshold switch (OTS),^13^, ^14^, ^15^, ^16^ metal–insulator transition (MIT),^17^, ^18^, ^19^, ^20^ mixed ionic–electronic conduction (MIEC),^21^, ^22^ field‐assisted superlinear threshold (FAST),^23^, ^24^ Schottky diodes,^25^, ^26^ multilayer oxide/nitride junctions,^27^, ^28^, ^29^, ^30^, ^31^, ^32^ etc. However, most of these selectors are still suffering from high leakage current issue, which would inevitably limit high density integration of X‐point arrays. Recently, a new type of metal‐filament threshold switch (TS), consisting of Ag (or Cu) as active electrode or dopant in solid electrolytes, has been demonstrated on low leakage characteristic and draws increasing attentions due to its high performance, simple structure, easy integration, and excellent compatibility with conventional CMOS technology.^33^ Different material system/device structures of metal‐filament TS devices have been proposed, as summarized in Table S1 (Supporting Information). Most of them have demonstrated either unidirectional or bidirectional characteristics but had limited on‐state currents and still need additional electroforming operations. It should be noted that the volatile TS would typically transit to a nonvolatile memory switch (MS) when the applied compliance current (*I*_cc_) is larger than 10 µA, as a result of stable filament growth.^34^, ^35^ Necessarily, selector devices need large on‐state current to provide sufficient current for memory operations, especially for the RESET operation. For instance, Panasonic, one of the few companies who are selling RRAM products, reported that TaO*_x_*‐based RRAM needs the RESET driving current (*I*_RESET_) close to 200 µA.^36^, ^37^ Besides, truly electroforming‐free characteristic is important for selectors. The initial electroforming process could usually lead to large device‐to‐device variations, and even worse, induce permanent damages to themselves in the 1S1R cells due to the applications of large voltages and currents.

Here, we present a novel strategy to achieve sufficient large RESET current (≈2.3 mA) by introducing the Ag nanodots/HfO_2_‐based bidirectional threshold switch (AND‐TS). This truly electroforming‐free selector demonstrates outstanding on‐state current, extremely high on/off ratio beyond 10^9^, ultralow leakage current below 1 pA, very steep slope of 0.65 mV dec^−1^, and good thermal stability up to 200 °C. Furthermore, the 1S1R integrated device consisting of AND‐TS and TaO*_x_*‐based resistive random access memory (RRAM) exhibits significant suppression of leakage currents and excellent performances in SET/RESET operations with a very high endurance exceeding 10^8^ cycles.

## 2 Results and Discussion

**Figure 1:**
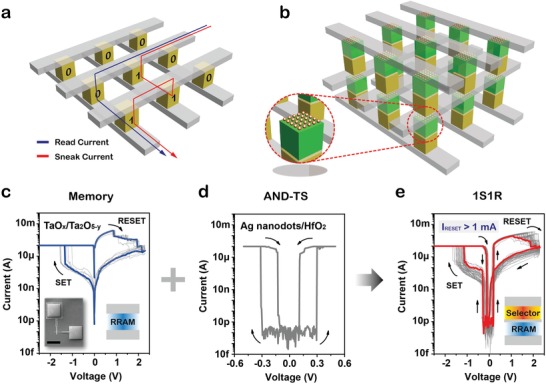
Cross‐point (X‐point) memory in one‐selector‐one‐resistor (1S1R) configuration. a) Schematic illustration of sneak current in 2D X‐point memory cells during read operation. “0” stands for off‐state, while “1” stands for on‐state. b) Schematic illustration of 3D X‐point memory with integration of ordered Ag nanodots based threshold switch (AND‐TS) and memory in 1S1R configuration. Red circle is the AND‐TS stack schematic of ordered Ag nanodots/HfO_2_ based selector. c) Repeated cycling *I*–*V* characteristics of TaO*_x_*/Ta_2_O_5−_ *_y_* bilayer RRAM. The inset shows SEM image of X‐point RRAM (left) and schematic illustration of RRAM stack structure (right). Scale bar: 100 µm. d) *I*–*V* characteristic of AND‐TS in X‐point structure. e) *I*–*V* characteristics of 1S1R integrated device in 50 DC voltage sweep cycles, exhibiting ultralow leakage currents (≈pA) at low voltages. The RESET currents (*I*_RESET_) are larger than 1 mA (maximal *I*_RESET_ ≈ 2.3 mA). The inset is schematic illustration of the 1S1R device stack structure.

The presence of sneak current paths is one of the most critical issues in X‐point architecture due to the extremely parallel geometry. **Figure** **1**a schematically shows a typical 2D X‐point array, where the sneak current through the on‐state (mark “1”) neighboring memory cells (red line path) could disturb the read operation from the target cell (blue line path). When the array size is getting larger, the leakage issue is getting even worse. Large sneak currents would increase total power consumption. Without any doubt, introducing a selector in series with memory cell is a very effective solution for the sneak current problem.^4^, ^5^ We propose a high‐performance selector, AND‐TS, that could be integrated with resistive memories, including but not limited to RRAM, in 2D or 3D X‐point structure to allow further density increase, as illustrated in Figure 1b.

The DC *I*–*V* characteristics of TaO*_x_*/Ta_2_O_5−_ *_y_* bilayer RRAM, AND‐TS selector, and their 1S1R integrated device are depicted in Figure 1c–e, respectively. The bilayer memory shows nonvolatile bipolar memory switching between a high resistance state (HRS) and a low resistance state (LRS) with *V*_SET_ = −1.4 V and *V*_RESET_ = 2.3 V (Figure 1c). Meanwhile, the AND‐TS is a bidirectional switching device, rather than the previously reported unipolar devices under large current compliance (*I*_cc_) conditions,^38^, ^39^ and shows volatile threshold switching with ultralow leakage current of ≈1 pA and high on‐state current of 100 µA under current compliance (Figure 1d). Through 1S1R integration, the sneak path current is greatly suppressed by the high‐performance selector, as presented in Figure 1e. The current of the 1S1R device maintains at ultralow ≈pA level when the value of applied voltage (*V*_a_) is smaller than that of threshold voltage (*V*_th_), while the current increases abruptly when the value of *V*_a_ is larger than *V*_th_. When AND‐TS turns on (|*V*_a_| > |*V*_th_|), TaO*_x_*‐based RRAM can perform effective SET and RESET operations at normal driving current level. More impressively, the 1S1R device exhibits sufficiently large *I*_RESET_ with ≈2.3 mA as the highest level during *I*–*V* sweeping measurements.

**Figure 2:**
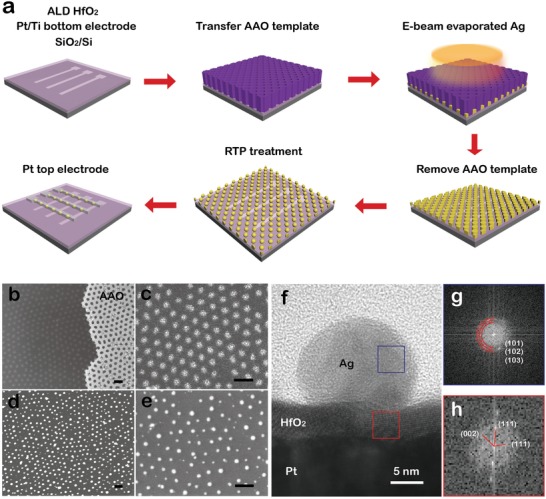
Device fabrication process and morphology characterization. a) Schematic illustration of AND‐TS fabrication process. b–e) SEM images of Ag nanodots morphology in the process (scale bar: 200 nm). b) Ordered Ag nanodots (left) fabricated through ultrathin AAO template (right). c) Enlarged SEM image of Ag nanodots before rapid thermal processing (RTP) treatment. d) SEM image of ordered Ag nanodots after RTP treatment. e) Enlarged SEM image of Ag nanodots after RTP treatment. f) STEM image of RTP‐treated Ag nanodot/HfO_2_ device stack layers, and the corresponding fast Fourier transform (FFT) patterns of g) Ag and h) HfO_2_.

As it is well known, TS behavior can be typically found at a low compliance current (*I*_cc_ = 10 µA) in most of metal‐filament TS selector devices, including the Ag thin film/HfO_2_‐based TS device (shown in Figure S1a, Supporting Information). This phenomenon can be attributed to the formation of very thin nanoscale Ag filament.^34^ However, the nanoscale Ag filament could further grow into large and stable one when the compliance current is over 10 µA, probably leading to nonvolatile MS transition.^34^ When compared to active metal (Ag or Cu) thin film being the electrode in TS devices, metallic nanoparticles (e.g., nanodots) as the active electrode could avoid excessive active metal atoms migration through the solid electrolyte during operations,^40^ which could be in favor of very thin metal filament formation and reduce the stability of the filament. An ultrathin anodic aluminum oxide (AAO) template^41^, ^42^, ^43^, ^44^ as pattern mask is applied to fabricate Ag nanodots on the prepared substrate followed by electron beam evaporation and rapid thermal processing (RTP). The fabrication process of AND‐TS is schematically illustrated in **Figure** **2**a and the details are also described in the Experimental Section. By adopting the ultrathin AAO template, highly ordered Ag nanodots array can be successfully obtained, and the corresponding Ag nanodots morphology before and after RTP treatment in the fabrication process are depicted in scanning electron microscope (SEM) images of Figure 2b–e, respectively. According to the cross‐sectional scanning transmission electron microscopy (STEM) image of the AND‐TS device stacks in Figure 2f, spherical Ag nanodot, HfO_2_ dielectric layer, and Pt electrode layer can be clearly observed. The Ag nanodot is polycrystalline, and the HfO_2_ layer shows the presence of crystalline phase, as the corresponding fast Fourier transform (FFT) images, respectively, shown in Figure 2g,h.

**Figure 3:**
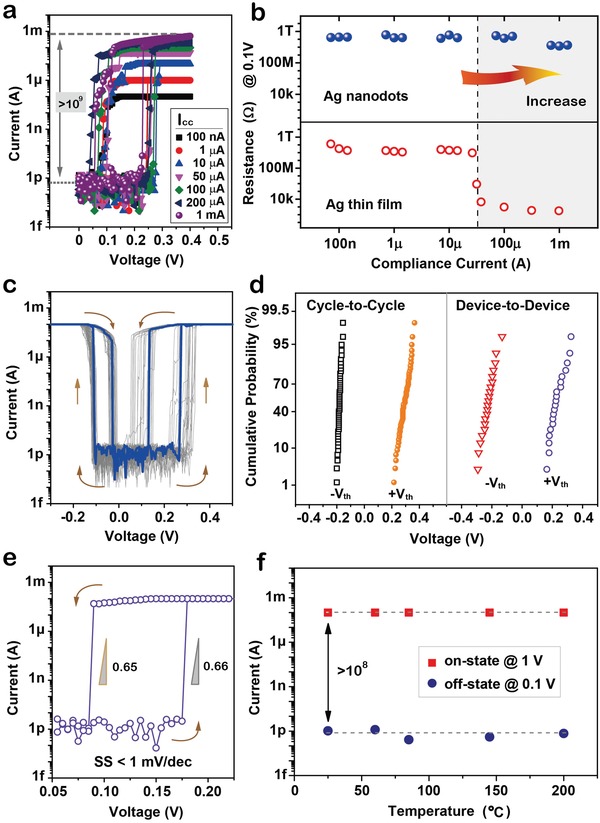
Excellent selector performance of AND‐TS. a) Threshold switching behavior of AND‐TS at different compliance current (*I*_cc_) from 100 nA to 1 mA, showing extremely high selectivity (on/off ratio) over 10^9^. b) Read resistance following turn‐on operation at increasing compliance currents for TS devices with RTP‐treated Ag nanodots (top) and Ag thin film (bottom) as active electrode. The device with RTP‐treated Ag nanodots (known as AND‐TS) can improve on‐state current up to 1 mA. c) Bidirectional threshold switching behavior of AND‐TS in 50 DC voltage sweep cycles. d) Cumulative probability of turn‐on voltage (threshold voltage, *V*_th_) as cycle‐to‐cycle (left) and device‐to‐device (right). e) AND‐TS has an extremely small turn‐on/turn‐off switching slope of <1 mV dec^−1^ (turn‐on: 0.66 mV dec^−1^; turn‐off: 0.65 mV dec^−1^). f) Thermal stability of AND‐TS at elevated temperatures. AND‐TS can still have high reliable current changes in large on/off ratio (>10^8^, under current compliance) although its work temperature varies from 25 to 200 °C.

Apparently, the volatile threshold switching behavior of AND‐ TS with a range of compliance currents from 100 nA to 1 mA is clearly shown in **Figure** **3**a. Meanwhile, the AND‐TS can work very well at different compliance current levels without initial electroforming operation. The truly electroforming‐free behavior could be attributed to an enhanced electric field induced by Ag thermal diffusion into dielectric layer, herein the embedded Ag nanoparticles can act as electric field concentrators,^45^ as the electric field simulation results shown in Figure S2 (Supporting Information). It should be noted that the AND‐TS can conduct a surprisingly large on‐state current while still retaining volatile threshold switching behavior. The *V*_a_ on the top electrode (Ag nanodots/Pt) is forward swept from 0 to +0.4 V and then reverse back to 0 V. The AND‐TS turns into on‐state from off‐state at a positive threshold voltage between 0.23 and 0.28 V, at which point the current rises rapidly to the limit of current compliance. Particularly, the on‐state current of the AND‐TS can apparently reach 680 µA under a compliance current of 1 mA, which exhibits self‐compliance behavior at a low voltage due to the limited cross‐sectional area of Ag filament.^46^ Indeed, the on‐state current level will be further improved when applied an increased voltage. The on‐state of AND‐TS is kept as *V*_a_ is sufficiently larger than *V*_th_, while the device transits to the off‐state as *V*_a_ reduces to below hold voltage (*V*_hold_). Moreover, read resistances at the voltage of 0.1 V for the TS devices based on Ag nanodots (RTP) and Ag thin film are, respectively, shown in Figure 3b, as *I*_cc_ increasing from 100 nA to 1 mA. The TS device with Ag nanodots exhibits a highly reliable volatile TS behavior even with the compliance current up to 1 mA, but the TS device with Ag thin film only works under a smaller compliance current of 10 µA; that is, the AND‐TS can increase the on‐state current level to more than two orders of magnitude, even when compared with other metal‐filament TS devices.^38^, ^39^, ^47^

Figure 3c illustrates 50‐cycle DC sweep *I*–*V* characteristics of AND‐TS at a large *I*_cc_ of 100 µA. After 50 cycles, the AND‐TS showed no obvious deterioration, and the off‐state resistance of the AND‐TS is about 10^11^ Ω at a read voltage of 0.1 V, representing an ultralow leakage current below 1 pA. Figure 3d shows the cycle‐to‐cycle (50 cycles) and device‐to‐device (20 devices) distribution of *V*_th_ for the AND‐TS, presenting tight distribution and good switching uniformity. Notably, the AND‐TS yields much steeper turn‐on and turn‐off switching slope (SS) of 0.66 and 0.65 mV dec^−1^ (both <1 mV dec^−1^), respectively, as shown in Figure 3e, which are significantly better than the previously reported.^39^, ^48^, ^49^ Moreover, the AND‐TS exhibits comparable fast switching speeds^39^ (see Figure S3, Supporting Information), and can switch for more than 10^8^ cycles, demonstrating an excellent endurance of the selector device (Figure S4, Supporting Information). Additionally, the AND‐TS shows a good temperature stability up to 200 °C (Figure 3f).

**Figure 4:**
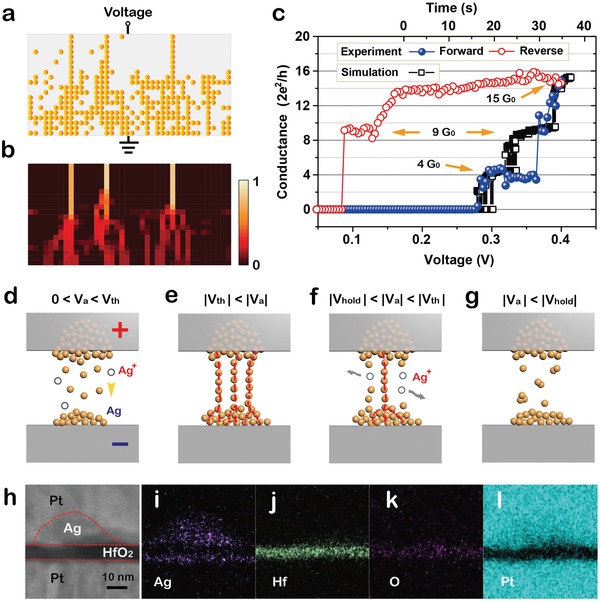
Mechanism of AND‐TS. a) Simulation for the formation of multiple weak Ag filaments with applied voltage (*V*_a_ = 0.4 V). b) Simulation for the corresponding conductive paths of multiple weak Ag filaments in normalized current. c) Conductance quantization characteristics of the AND‐TS device in experiment (forward voltage sweep: blue; reverse voltage sweep: red) and simulation (black) results. d–g) Schematic illustration for the mechanism of AND‐TS based on Ag filament formation/rupture under different voltage bias. h) HAADF‐STEM cross‐sectional image of AND‐TS stack layers. i–l) Energy‐dispersive X‐ray spectroscopy (EDS) mapping of Ag, Hf, O, and Pt elements in the device stack layers, respectively, after one‐time positive voltage sweeping.

The switching dynamics are strongly dependent on the morphology of conductive filaments.^33^ For the TS device with Ag thin film as active electrode, the volatile TS characteristics of the device can transit to the nonvolatile MS characteristics as *I*_cc_ larger than 10 µA, due to excessive Ag atoms migration into the solid electrolyte inducing stable metal‐filament formation (Figure S1a, Supporting Information); The TS device with Ag nanodots (no RTP) as active electrode can only cycle a few times at a large compliance current of 100 µA, which indicates a more stable Ag filament is gradually formed after switching in a few cycles (Figure S1b, Supporting Information). However, both types of devices require additional electroforming operations. Furthermore, the truly electroforming‐free AND‐TS, which is featured with RTP‐treated Ag nanodots as active electrode, demonstrates superior selector performances with sufficient large on‐state current and extremely high selectivity (Figure S1c, Supporting Information). According to experimental results and Monte Carlo simulations (the flow chart is shown in Figure S5, Supporting Information), the mechanisms of AND‐TS are believed to be the formation and rupture of multiple weak Ag filaments under different voltage bias, as illustrated in **Figure** **4**. The ultrathin AAO template makes Ag nanodot structure, restricting the silver atoms/clusters distribution at the interface of top electrode and HfO_2_ and further inhibiting excessive migration of silver. In addition, RTP treatment could contribute to some Ag atoms diffusion into the HfO_2_ matrix, and the Ag atom would accumulate on the interface of bottom electrode/HfO_2_. The formation dynamics of multiple weak Ag filaments with applied voltage of 0.4 V are simulated in Figure 4a, and the corresponding current conductive paths are illustrated in Figure 4b. The existence of prediffused Ag atoms in HfO_2_ matrix would induce multiple channels that generate larger intensities in electric field along the directions of Ag nanoparticles distribution, as the electric field distribution simulated by COMSOL in Figure S2c (Supporting Information). Moreover, Ag nanodot with RTP treatment could be helpful for Ag atoms in limited quantity preferentially acting as interstitial dopants in HfO_2_.^48^ Indeed, the formation/rupture of Ag filaments is accompanied with the doping/dedoping procedures of Ag.^34^ The existence of Ag at the doping state in HfO_2_ would enable the formation of conductive filaments.^34^, ^48^ It is remarkable that multiple weak Ag filaments, which could preferentially form as applying a lower voltage bias, result in a reasonable large on‐state current of >500 µA, as proved by the simulation results. More importantly, we can directly observe the jump steps of quantized conductance (*G*_0_ = 2*e* ^2^/*h*) that may provide experimental evidence for the formation/rupture procedures of multiple weak Ag filaments during switching, as illustrated in Figure 4c. The electrical conductance *G* (*G* = *I*/*V*) of AND‐TS increases abruptly by the steps of integer or half integer of *G*_0_, when the AND‐TS turns from off‐state (≈1 pA) to on‐state (*I*_cc_ = 500 µA) as the voltage forward increasing from 0.28 to 0.4 V (blue). Each current jump, which also corresponds to quantized conductance, is connected to an Ag filament that will bridge the solid electrolyte and establish a galvanic contact.^46^ In Figure S6 (Supporting Information), the conductance histograms of quantized states both for the simulation and experiment are statistically analyzed. Meanwhile, the quantized conductance states for three big jumps arriving at 4, 9, and 15 G_0_, respectively, may correspond to three very thin nanoscale Ag filaments growth. Afterward, the conductance G reduces by integer or half integer of *G*_0_ down to 9 *G*_0_ and finally turns into off‐state, due to the Gibbs–Thomson effect or nanobattery effect occurred in the very thin Ag filaments,^34^, ^39^ when the voltage reverse sweeping back, which is definitely presenting the spontaneous rupture of the nanoscale multiple Ag filaments contact. In addition, the formation of Ag filaments can be determined by the compliance currents and applied voltages. And the case under an *I*_cc_ of 100 µA is shown in Figure S7 (Supporting Information). Moreover, the conductance would increase to a higher quantized state with the limited Ag filament conductive paths under a small voltage bias, but the current actually cannot reach the limit of compliance current, hence the self‐compliance behavior can be typically observed. Similarly, the AND‐TS can achieve a large on‐state current below the compliance current of 1 mA (at *V*_a_ = 0.4 V), which may contribute to few nanoscale Ag filaments growth with limited cross‐sectional area.

The formation and rupture procedures of multiple weak Ag filaments can be controlled by the applied voltage, which are schematically illustrated in Figure 4d–g. When applying *V*_a_ on the top electrode (0 < *V*_a_ < *V*_th_), the oxidized Ag^+^ ions from the top electrode or the diffused Ag penetration could be chemically reduced to Ag atoms or as‐clusters within the dielectrics, due to the low ion mobility and low redox reaction rate of Ag^+^ ions in HfO_2_ 39, 50 (Figure 4d). When *V*_a_ reaches the value of *V*_th_ (|*V*_th_| < |*V*_a_|), the reduced Ag atoms can align to form multiple atomic chains (weak Ag filaments) in HfO_2_ as a result of larger concentrations of electric field channels (Figure 4e), which is surely align with quantized conductance changes in Figure 4b. Subsequently, when *V*_a_ decreases to the value of *V*_hold_ (|*V*_hold_| < |*V*_a_| < |*V*_th_|), some parts of multiple weak Ag filaments happen spontaneous rupture (Figure 4f) due to the repulsive stress between the Ag^+^ ions without enough energy to keep Ag atomic chains in reduction state, which are determined by the Gibbs–Thomson effect. Besides, nonequilibrium of chemical potential induced nanobattery effect may also assist the spontaneous rupture of Ag filaments.^51^ When *V*_a_ further decreases below *V*_hold_ (|*V*_a_| < |*V*_hold_|), the device turns into off‐state finally as a result of Ag filaments in complete rupture state. Obviously, some Ag atoms/clusters distribute at both interfaces of electrode/HfO_2_, which provides the possibility for bidirectional operations, according to high angle annular dark field STEM (HAADF‐STEM) cross‐sectional image of AND‐TS stack layers and the corresponding energy‐dispersive X‐ray spectroscopy (EDS) mapping of Ag, Hf, O, and Pt elements in Figure 4h–l.

**Figure 5:**
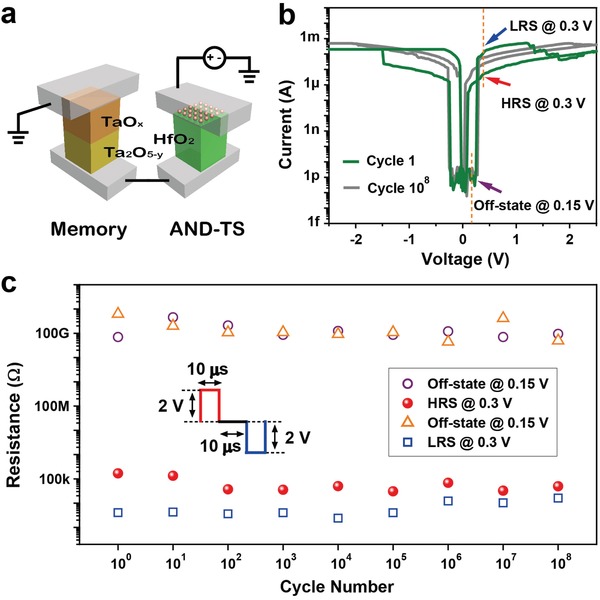
1S1R configuration in X‐point devices. a) Schematic illustration for 1S1R integration with resistive memory (TaO*_x_*/Ta_2_O_5−_ *_y_*) and AND‐TS. b) *I*–*V* characteristics of the integrated 1S1R device before and after 10^8^ cycles. c) Excellent endurance during over 10^8^ pulse measurement for 1S1R configuration. The waveform employed in the endurance measurement which consists of 10 µs pulses with amplitudes of ±2 V for RESET/SET operations, and the time interval between switching pulses is 40 µs. The read operations are conducted by applying two voltage biases (0.15 and 0.3 V) simultaneously after a certain number of pulses testing.

Getting the AND‐TS series with a TaO*_x_*/Ta_2_O_5−_ *_y_* bilayer RRAM is schematically illustrated in **Figure** **5**a. To increase *V*_a_ in negative direction, the AND‐TS comes to turn on abruptly at *V*_th_ of about −0.25 V, and then followed by the SET transition of the memory at about −1.5 V. The subsequent reading operation verifies the LRS of the memory. During the positive voltage sweep, the AND‐TS turns on at about +0.25 V followed by a gradual RESET transition of the memory. The conductance quantization characteristic of the 1S1R device in positive voltage sweep is illustrated in Figure S8 (Supporting Information). The quantized conductance increases abruptly to 7.5 *G*_0_ when the AND‐TS is turning on, and further increases from 8 to 12.5 *G*_0_ by the steps of integer or half integer of *G*_0_. And then followed by RESET voltage sweeping, the conductance comes to decrease. In fact, the maximum conductance of the 1S1R device is confined to the quantized states of AND‐TS when RRAM is in LRS. That is, an enough large quantized conductance of AND‐TS, which corresponds to a sufficient large on‐state current, will maintain very reliable RESET operations for the 1S1R device. In addition, the 1S1R device with AND‐TS selector can achieve large read margins and no significant degradation even in a large array size of 1Mb, when compared with 1R and other 1S1R devices,^13^, ^52^, ^53^ as the simulation shown in Figure S9 (Supporting Information). More impressively, this 1S1R device demonstrates an extremely high endurance for SET/RESET operations after 10^8^ cycles at program/erase pulse voltage (Figure 5b,c), which is a superior result for 1S1R configuration.^24^ Truly, the AND‐TS exhibits comparable stable threshold/hold voltages of +0.25 V/+0.05 V and −0.25 V/−0.05 V before and after more than 10^8^ times pulse endurance testing (as shown in Figure 5b), although the RRAM suffers some degree of degradation during endurance testing with lower on/off windows (Figure 5c).

## 3 Conclusion

In summary, the sneak path currents can be effectively suppressed by introducing a novel bidirectional threshold switch, which is featured with high‐ordered Ag nanodots. The AND‐TS, without additional electroforming operation, has sufficiently large on‐state current, extremely high on/off ratio beyond 10^9^, very steep slope of 0.65 mV dec^−1^, and good thermal stability up to 200 °C. The superior AND‐TS performances are attributed to RTP‐treated Ag nanodots, which could contribute to form multiple weak Ag filaments and then spontaneous rupture as applied voltage reduced. Furthermore, the 1S1R device exhibits significant suppression of leakage current and shows excellent characteristics in SET/RESET operations with a high endurance over 10^8^ cycles, where the maximum *I*_RESET_ of ≈2.3 mA was obtained during the *I*–*V* sweeping tests. In addition, the AND‐TS selector has suitable *V*_th_ by controlling the thickness of HfO_2_ layer (Figure S10, Supporting Information) to couple with resistive memory in 1S1R configuration (see Figure S11, Supporting Information). Looking forward, the AND‐TS, featuring sufficient on‐state current, superior *I*–*V* nonlinearity, truly electroforming‐free, and high endurance, may thus lead to innovative applications in circuits and systems, especially for X‐point memory applications.

## 4 Experimental Section

*Fabrication of AND‐TS*: Device stacks (size: 5 × 5 µm^2^) were prepared on a substrate of Si wafer with 200 nm thermal oxidized SiO_2_. Bottom electrode was patterned by photolithography (Cannon PLA 550), sputtered (Kurt J. Lesker LAB18) with 5 nm Ti and 50 nm Pt, and then lift‐off the thin films. 5 nm HfO*_x_* thin film was prepared by atomic layer deposition (ALD, Beneq TFT 200) at 200 °C. An ultrathin anodic aluminum oxide (AAO) template was transferred onto the above prepared layers, followed by e‐beam evaporated (Denton, explorer 128) Ag thin film. After the AAO template removed, the sample was treated by the rapid thermal processing (RTP) at 500 °C for 30 s. Finally, 40 nm Pt thin film as top electrode, patterned and deposited by lithography and sputter, respectively.

*Fabrication of TaO_x_‐Based RRAM*: TaO*_x_*/Ta_2_O_5−_ *_y_* bilayer stacks (40 nm/20 nm) were sputtered with different oxygen ratio atmosphere. The Pt/TaO*_x_*/Ta_2_O_5−_ *_y_*/Pt cross‐point memory stack with an area of 3 × 3 µm^2^ consisting of 50 nm Pt bottom/top electrodes patterned by photolithography. In addition, the 1S1R device was measured by using AND‐TS selector connected to the TaO*_x_*‐based RRAM.

*Microstructural Characterizations*: SEM images were obtained by Hitachi SU8020. TEM cross‐sectional samples were prepared using FIB (FEI Helios). HAADF‐STEM images and EDS mapping were obtained by STEM (FEI Tecnai F20).

*Electrical Measurements*: DC voltage/current sweeps were measured by Agilent B1500A semiconductor device parameter analyzer. The delay and relaxation speed of AND‐TS were performed by Agilent B1530A Waveform Generator/Fast Measurement Unit (WGFM) and Agilent B1500A semiconductor device parameter analyzer. The endurance measurements of AND‐TS or 1S1R device were carried by a test system consisting of Agilent B1110A pulse/pattern generator, Agilent B1500A semiconductor device parameter analyzer, and Agilent B2201A 14ch low leakage switch mainframe.

*Simulation*: Atomic stochastic simulation was introduced to self‐consistently investigate the microscopic processes of Ag migration.^54^ The flow chart for Monte Carlo simulation method is schematically depicted in Figure S5 (Supporting Information). The distribution of electrical potential and current density can be solved by the resistor network model. The probabilities of generation (oxidation/reduction: Ag → Ag^+^ + e^−^; Ag^+^ + e^−^ → Ag) of Ag atoms and migration of Ag^+^ can be given by *P* = *f*∙exp((−(*E* − ∆ϕ))/(*k*_B_ *T*)), where *f* is the vibration frequency, *E* is activation energy of oxidation/reduction or hopping barrier of Ag^+^, respectively, and ∆ϕ is barrier height reduction induced by electric field. By applying a voltage bias on the active electrode, Ag atoms redistribution and the corresponding electric conductance can be achieved via simulation. In addition, the simulation of 3D space was mapped to the 2D plane in order to simplify the calculation procedures. Besides, electric field distribution was conducted by COMSOL simulation using the Electrostatics module.

## Conflict of Interest

The authors declare no conflict of interest.

## Supporting information

Supplementary
