# Supplementary material for: A Threshold Switching Selector Based on Highly Ordered Ag Nanodots for X‐Point Memory Applications
Source: Adv Sci (Weinh). 2019 Apr 2;6(10):1900024. doi: 10.1002/advs.201900024 (PMC6524079; doi:10.1002/advs.201900024)
Supplement: Supplementary file 1 — Supplementary [file ADVS-6-1900024-s001.pdf]

Copyright WILEY-VCH Verlag GmbH & Co. KGaA, 69469 Weinheim, Germany, 2019.

ADVANCED SCIENCE
Open Access

Supporting Information

for *Adv. Sci.,* DOI: 10.1002/advs.201900024

A Threshold Switching Selector Based on Highly Ordered Ag Nanodots for X-Point Memory Applications

Qilin Hua, Huaqiang Wu, Bin Gao,* Meiran Zhao, Yujia Li, Xinyi Li, Xiang Hou, Meng-Fan (Marvin) Chang, Peng Zhou,* and He Qian*

Copyright WILEY-VCH Verlag GmbH & Co. KGaA, 69469 Weinheim, Germany, 2019.

Supporting Information

Threshold Switching Selector based on Highly-ordered Ag nanodots for X-point Memory Applications

Qilin Hua, Huaqiang Wu, Bin Gao*, Meiran Zhao, Yujia Li, Xinyi Li, Xiang Hou, Meng-Fan (Marvin) Chang, Peng Zhou*, He Qian*

Dr. Q. Hua, Prof. H. Wu, Prof. B. Gao, M. Zhao, Y. Li, Dr. X. Li and Prof. H. Qian
Institute of Microelectronics, Tsinghua University, Beijing, 100084, China
E-mail: wuhq@tsinghua.edu.cn; gaob1@tsinghua.edu.cn

X. Hou, Prof. P. Zhou
State Key Laboratory of ASIC and System, School of Microelectronics, Fudan University, Shanghai 200433, China
Email: pengzhou@fudan.edu.cn

Prof. M.-F. Chang
Department of Electrical Engineering, National Tsing Hua University, Taiwan

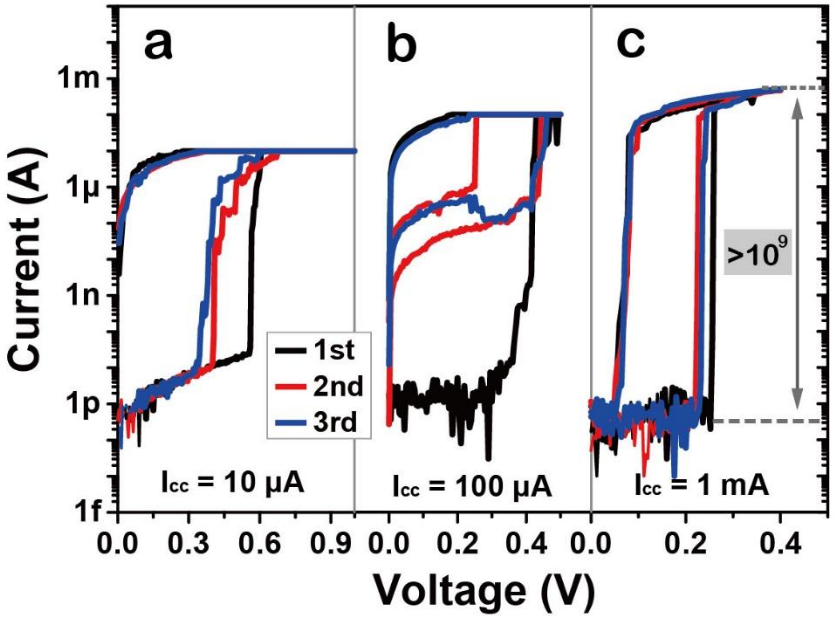

Figure S1. Three consecutive IV sweep curves of the Ag-filament TS devices (device structure: Pt/Ag/HfO_2/Pt) with Ag thin film (a), Ag nanodots without RTP (b) and Ag nanodots with RTP (c), respectively. The TS device with Ag thin film has a limited on-state current of 10 μA; The TS device with Ag nanodots would turn TS behavior to MS behavior at I_{cc} = 100 μA in few cycles; Amazingly, the TS device with RTP treated Ag nanodots (AND-TS) shows good TS stability with an extremely high selectivity over 10^9 and ultralow leakage current below 1 pA.

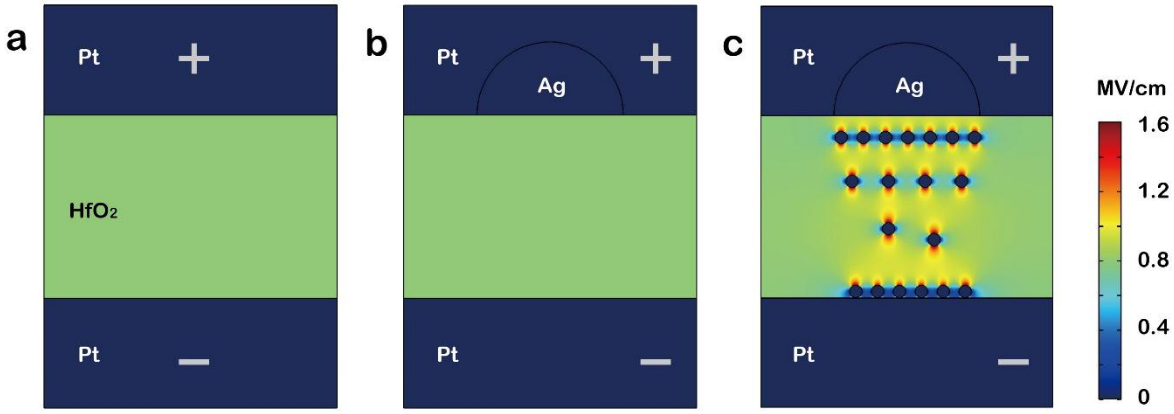

Figure S2. Electric field distribution in the TS devices based on Ag thin film (a), Ag nanodot (b) and RTP treated Ag nanodot (c) as active electrode by COMSOL simulation (using the *Electrostatics* module). As illustrated, the generated electric field for the TS device with RTP-treated Ag nanodot would increase by ~ 25% due to Ag thermal diffusion into dielectric layer, when compared with those devices based on thin film or Ag nanodot under the same voltage bias of 0.4 V. It may be the reason that the AND-TS does not need additional electroforming operation in advance. In addition, multiple Ag-filaments in the AND-TS would be induced to grow along the larger electric field distribution.

Model description:

Device structure: Pt/Ag nanodot/HfO_2/Pt

Parameter: HfO_2 ---- thickness: 5 nm; width: 8 nm; dielectric constant: 25.

Applied voltage: 0.4 V.

Electrostatics equations:

$$\nabla \cdot \mathbf{D} = \rho_V$$

$$\mathbf{E} = -\nabla V$$

where D is the electric displacement, ρ_V is the free charge density, E is the electric field, and V is the electric potential.

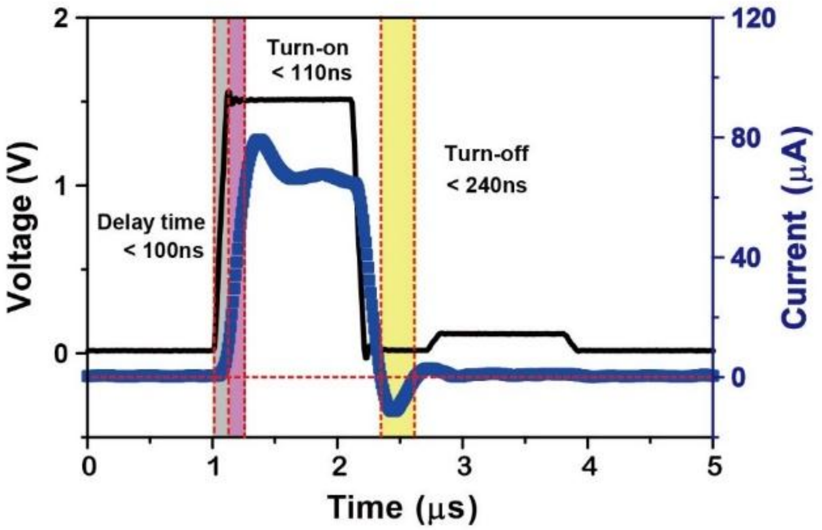

Figure S3. Switching speed measurement of AND-TS. The current changes (blue curve) are recorded as applying turn-on voltage (1 μs, 1.5 V) and read voltage (1 μs, 0.1 V) pulses (black curve). Delay time < 100 ns, turn-on time < 110 ns, and fast turn-off time < 240 ns.

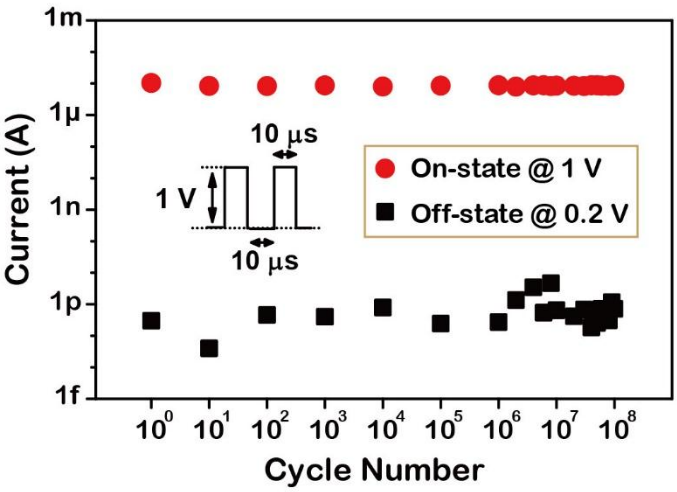

Figure S4. Endurance of AND-TS selector. The measurement is conducted with a series resistor of 81 kΩ. The waveform consists of a 10 μs pulse with an amplitude of 1 V for turn-on switching, and then followed by read voltages of 0.2 V and 1 V. Time interval between switching pulses is 20 μs.

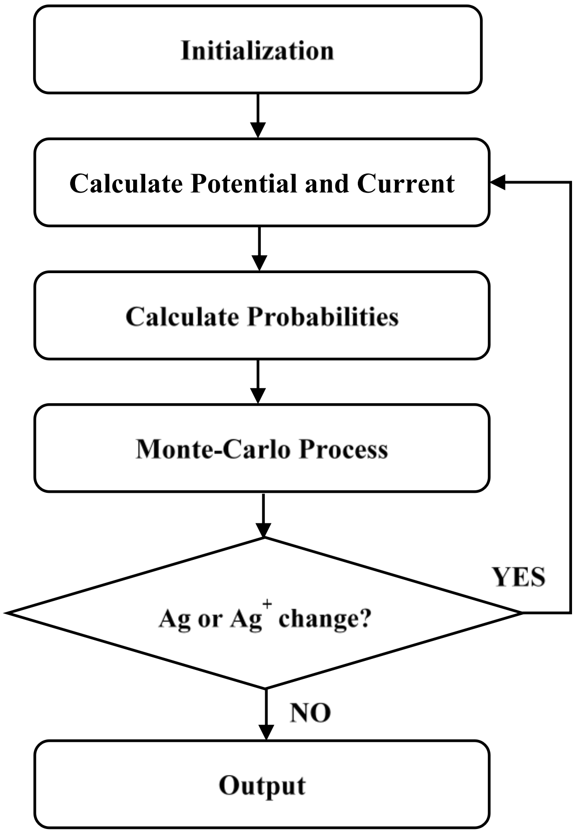

Figure S5. Schematic flow chart of the Monte Carlo simulation for Ag-filament formation.

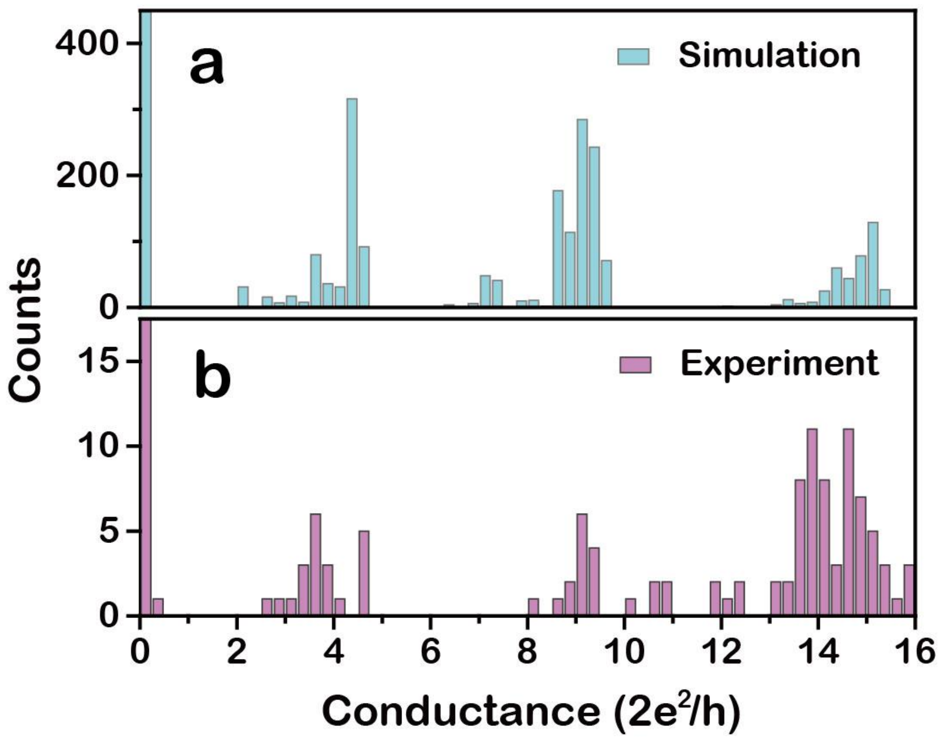

Figure S6. The corresponding conductance histograms of quantized states in the AND-TS shown in Fig. 4c. (a) simulation; (b) experiment. The quantized states are mainly centered on 4 G_0, 9 G_0 and 15 G_0, respectively, which may correspond to three very thin nanoscale Ag-filaments growth.

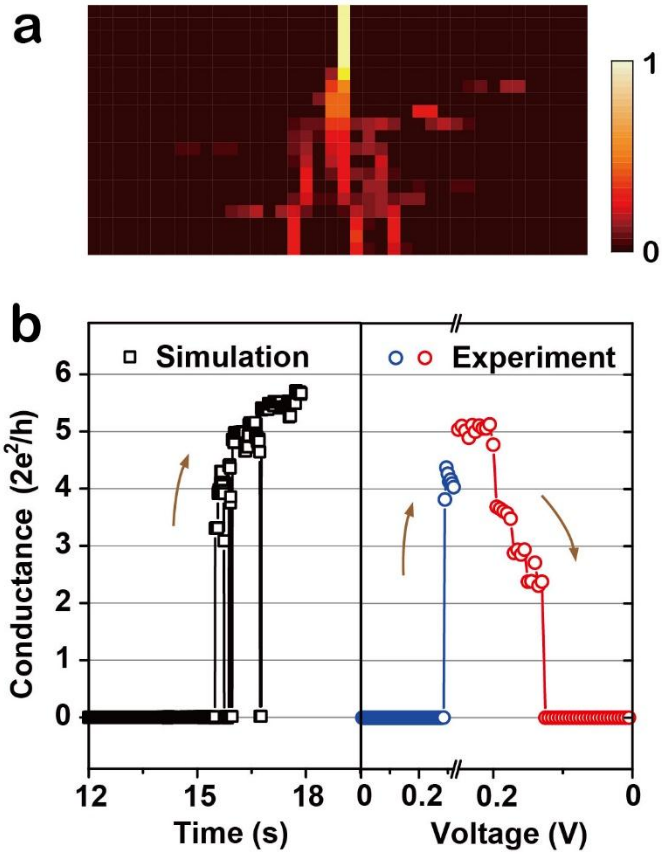

Figure S7. Ag-filament formation of AND-TS under a I_{cc} of 100 μA. (a) The normalized current distribution induced by the formed one weak Ag-filament. (b) The corresponding conductances in quantized states as one weak Ag-filament formation. And the galvanic contact procedures can be observed as the conductance jumps to 3 G_0, 4 G_0, and 5 G_0. (c) The conductance quantization characteristic of the AND-TS under a I_{cc} of 100 μA in experiment (forward voltage sweep: blue; reverse voltage sweep: red). The quantized conductance equally confines to 4 G_0 and 5 G_0, and simultaneously validate the formation and rupture procedures of Ag-filaments under the current compliance condition

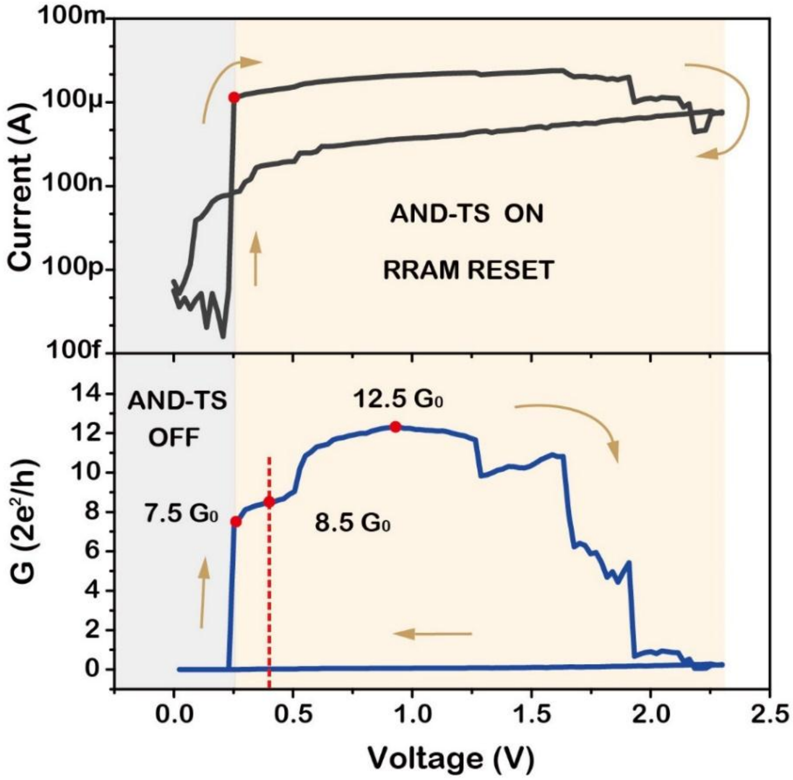

Figure S8. The conductance quantization characteristic of the 1S1R device. (a) I-V characteristic of the 1S1R device in RESET operation. (b) The corresponding conductances in quantized states of the 1S1R device in RESET operation. The red dashed line indicates the applied voltage of 0.4 V, and the corresponding quantized conductance is about 8.5 G_0.

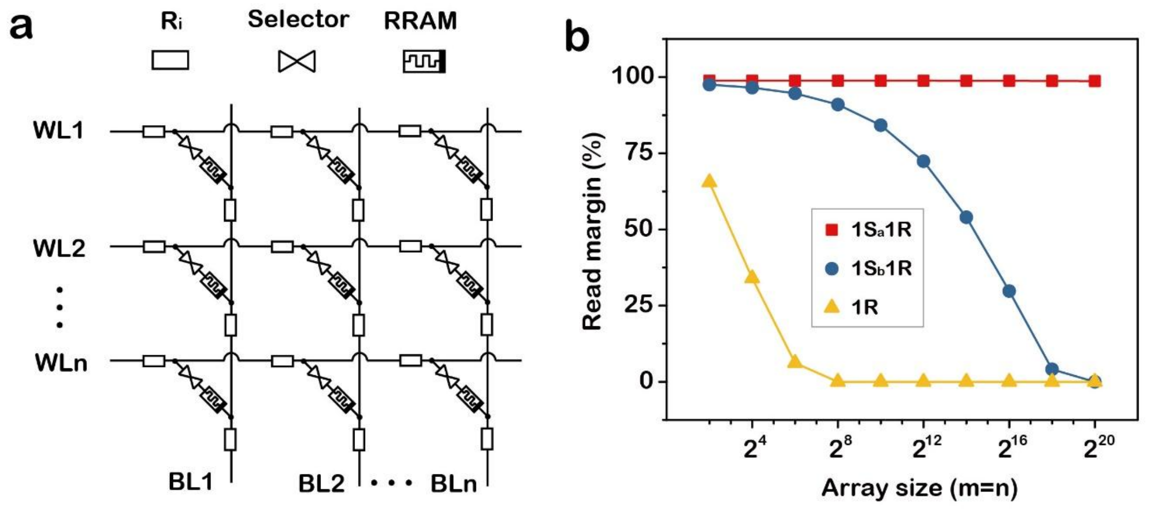

Figure S9. Simulation of the X-point memory array in 1S1R configuration. (a) Schematic of the 1S1R-based X-point array, consisting of wire resistors R_i, selector and RRAM. (b) Read margin analysis in the worst case condition for the 1R and two different 1S1R configurations. R: TaO_x/Ta_2O_{5-y} based RRAM; S_a: AND-TS selector; S_b: OTS (AsGeTeSiN) selector.[1]

Key parameters for the simulations

R_i = 0.5 Ω	R	S_a	S_b
Device	TaO_x/Ta_2O_{5-y}	AND-TS	OTS (AsGeTeSiN)
LRS (or R_{on})	10 kΩ	2 kΩ	10 kΩ
HRS(or R_{off})	1 MΩ	100 GΩ	2 MΩ
V_{th}	-	0.27 V	1.2 V
V_{hold}	-	0.05 V	0.8 V
V_{read}	0.5 V	0.5 V	2 V

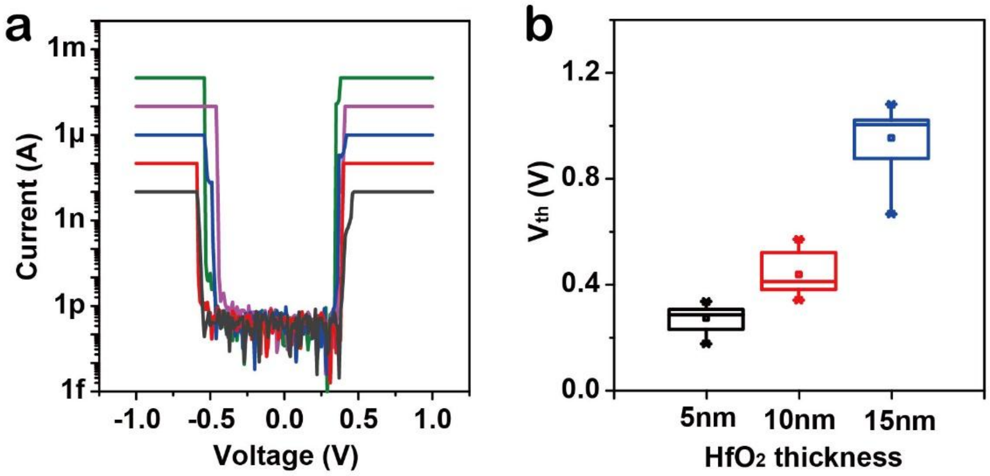

Figure S10. Controlled threshold voltage (V_{th}) of AND-TS with different thickness of HfO_2 layer. (a) I-V characteristics of AND-TS in forward sweeps (I_{cc}: 10 nA to 100 μA), which shows the possibility for the increased V_{th} as adding thickness of HfO_2 to 10 nm. (b) The relation between V_{th} and HfO_2 layer thickness. Error bars represent the variation of V_{th} in ten devices.

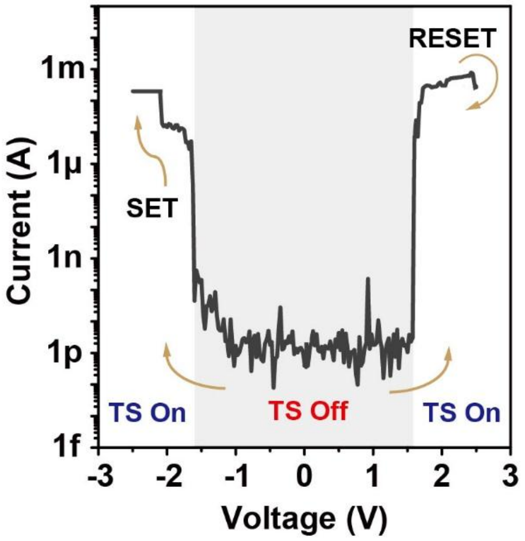

Figure S11. The I-V characteristic of 1S1R with AND-TS which has large V_{th} by increasing HfO_2 thickness (~20 nm).

Table S1. The comparison of threshold switching selectors in different device structures.

Device Structure	Switch mode	I_{on}	I_{off}	On/off ratio	V_{th}	Switch Slope	Relax time	Endurance	Temperature stability	1S1R	Ref.
TiN/AsTeGeSiN/TiN (OTS)	Bipolar	>100µA 11MA/cm^2	~0.1µA	10^3	±1V	-	2ns	10^8	400℃	Yes	[1]
W/NiO$_y$/NbO$_x$/NiO$_y$/W (MIT)	Bipolar	>1mA	~0.1µA	5400	±1.4V	-	2ns	10^8	180℃	Yes	[2]
TE/SLT/BE (FAST)	Bipolar	100µA (I_{cc})	~10pA	10^{10}	±1V	<5mV/dec	50ns	10^8	125℃	Yes	[3]
Ag/TiO$_2$/Pt	Bipolar	10µA (I_{cc})	~1pA	10^7	0.24V, -0.5V	<5mV/dec	-	-	-	No	[4]
Pt/HfO$_2$/ Cu:HfO$_2$/Cu	Uipolar	10µA (I_{cc})	~1pA	10^7	~0.4V	-	-	>10^{10}	-	No	[5]
Pt/Cu$_2$O/Ag:Cu$_2$O /Cu$_2$O/Pt	Bipolar	1µA	~1nA	10^3	±0.6V	-	-	-	-	No	[6]
Ag/a-Si:H/Pt	Bipolar	10µA (I_{cc})	~1pA	10^7	±0.8V	<5mV/dec	-	-	-	No	[7]
Cu/SiO$_2$/Pt	Unipolar	500µA (I_{cc})	~10pA	10^7	0.5~0.8V	-	-	-	-	No	[8]
Ag/ZrO$_2$/Pt	Bipolar	1mA (I_{cc})	~0.1nA	10^7	±0.15V	-	-	-	-	No	[9]
AgTe/TiN/TiO$_2$/Pt	Unipolar	100µA (I_{cc})	~1pA	10^8	0.5V	<5mV/dec	100ns	-	400℃	No	[10]
W/Cu$_2$S/W	Unipolar	10µA (I_{cc})	~100pA	10^5	0.3V	<5mV/dec	-	-	-	No	[11]
Ag/HfO$_2$/p-Si	Unipolar	100µA (I_{cc})	~10pA	10^7	1.6V	3mV/dec	67ns	10^8	90℃	Yes	[12]
Ag/SiO$_x$/C	Bipolar	50µA (I_{cc})	~1pA	>10^7	2V, -0.5V	-	-	-	-	Yes	[13]
Pd/Ag/HfO$_x$/Ag/Pd	Bipolar	100µA (I_{cc})	~1pA	10^8	±0.4V	1mV/dec	250ns	10^8	85℃	Yes	[14]
Pt/Ag:ZnO/Pt	Bipolar	100µA (I_{cc})	~0.1pA	>10^9	0.5V	5mV/dec	100ns	-	250℃	No	[15]
Pt/Ag nanodots/ HfO$_2$/Pt	Bipolar	>1mA	<1pA	>10^9	±0.25V	0.65mV/dec	240ns	>10^8	200℃	Yes	This work

REFERENCES

[1] M. J. Lee, D. Lee, S. H. Cho, J. H. Hur, S. M. Lee, D. H. Seo, D. S. Kim, M. S. Yang, S. Lee, E. Hwang, M. R. Uddin, H. Kim, U. I. Chung, Y. Park, I. K. Yoo, *Nat. Commun.* **2013**, *4*, 2629.

[2] J. Park, T. Hadamek, A. B. Posadas, E. Cha, A. A. Demkov, H. Hwang, *Sci. Rep.* **2017**, *7*, 4068.

[3] J. Sung Hyun, T. Kumar, S. Narayanan, W. D. Lu, H. Nazarian, presented at *2014 IEEE Int. Electron Devices Meet. (IEDM)* "3D-stackable crossbar resistive memory based on Field Assisted Superlinear Threshold (FAST) selector", San Francisco, CA, USA, 15-17 Dec., **2014**.

[4] J. Song, J. Woo, A. Prakash, D. Lee, H. Hwang, *IEEE Electron Device Lett.* **2015**, 36, 681.

[5] Q. Luo, X. Xu, H. Liu, H. Lv, T. Gong, S. Long, Q. Liu, H. Sun, W. Banerjee, L. Li, N. Lu, M. Liu, presented at *2015 IEEE Int. Electron Devices Meet. (IEDM)* "Cu BEOL compatible selector with high selectivity ($>10^7$), extremely low off-current (~pA) and high endurance ($>10^{10}$)", Washington, DC, USA, 7-9 Dec., **2015**.

[6] J. Song, A. Prakash, D. Lee, J. Woo, E. Cha, S. Lee, H. Hwang, *Appl. Phys. Lett.* **2015**, *107*, 113504.

[7] J. Yoo, J. Woo, J. Song, H. Hwang, *AIP Adv.* **2015**, *5*, 127221.

[8] W. Chen, H. J. Barnaby, M. N. Kozicki, *IEEE Electron Device Lett.***2016**, *37*, 580.

[9] G. Du, C. Wang, H. Li, Q. Mao, Z. Ji, *AIP Adv.* **2016**, *6*, 085316.

[10] J. Song, J. Park, K. Moon, J. Woo, S. Lim, J. Yoo, D. Lee, H. Hwang, presented at *2016 IEEE Int. Electron Devices Meet. (IEDM)* "Monolithic integration of AgTe/TiO_2 based threshold switching device with TiN liner for steep slope field-effect transistors", San Francisco, CA, USA, 3-7 Dec., **2016**.

[11] S. Lim, J. Yoo, J. Song, J. Woo, J. Park, H. Hwang, presented at *2016 IEEE Int. Electron Devices Meet. (IEDM)* "Excellent threshold switching device (I_{of} ~ 1 pA) with atom-scale metal filament for steep slope (< 5 mV/dec), ultra low voltage (V_{dd} = 0.25 V) FET applications", San Francisco, CA, USA, 3-7 Dec., **2016**.

[12] N. Shukla, B. Grisafe, R. K. Ghosh, N. Jao, A. Aziz, J. Frougier, M. Jerry, S. Sonde, S. Rouvimov, T. Orlova, S. Gupta, S. Datta, presented at *2016 IEEE Int. Electron Devices Meet. (IEDM)* "Ag/HfO_2 based threshold switch with extreme non-linearity for unipolar cross-point memory and steep-slope phase-FETs", San Francisco, CA, USA, 3-7 Dec., **2016**.

[13] A. Bricalli, E. Ambrosi, M. Laudato, M. Maestro, R. Rodriguez, D. Ielmini, presented at *2016 IEEE Int. Electron Devices Meet. (IEDM)* "SiO_x-based resistive switching memory (RRAM) for crossbar storage/select elements with high on/off ratio", San Francisco, CA, USA, 3-7 Dec., **2016**.

[14] R. Midya, Z. Wang, J. Zhang, S. E. Savel'ev, C. Li, M. Rao, M. H. Jang, S. Joshi, H. Jiang, P. Lin, K. Norris, N. Ge, Q. Wu, M. Barnell, Z. Li, H. L. Xin, R. S. Williams, Q. Xia, J. J. Yang, *Adv. Mater.* **2017**, *29*, 1604457.

[15] U.-B. Han, D. Lee, J.-S. Lee, *NPG Asia Mater.* **2017**, *9*, e351.

[16] B. Gao, B. Sun, H. Zhang, L. Liu, X. Liu, R. Han, J. Kang, B. Yu, *IEEE Electron Device Lett.***2009**, *30*, 1326.

[17] B. Gao, J. F. Kang, Y. S. Chen, F. F. Zhang, B. Chen, P. Huang, L. F. Liu, X. Y. Liu, Y. Y. Wang, X. A. Tran, Z. R. Wang, H. Y. Yu, A. Chin, presented at *2011 IEEE Int. Electron Devices Meet. (IEDM)* "Oxide-based RRAM: Unified microscopic principle for both unipolar and bipolar switching", Washington, DC, USA, 5-7 Dec. **2011**.
